# Simulation of vorticity wind turbines

**DOI:** 10.1016/j.heliyon.2020.e05155

**Published:** 2020-10-09

**Authors:** Paolo Sassi, Jorge Freiría, Mariana Mendina, Martin Draper, Gabriel Usera

**Affiliations:** IMFIA - UdelaR - J. Herrera y Reissig 565, Montevideo, 11300, Uruguay

**Keywords:** Energy, Mechanical engineering, Immersed boundary method, Computational fluid dynamics, Finite volume method, Vorticity wind turbines, Discrete element method

## Abstract

There are a wide variety of devices behaving essentially as flexible and elastic systems while interacting dynamically with fluids, usually water or air, under normal operating conditions. Interactions of this kind involve a double complexity of the dynamics, as the systems go through large deformation due to the flow actions, and simultaneously, the flow dynamics is strongly influenced by the shape adopted by the systems. The present research adapts mathematical methods, still new to the field, to represent ways of dealing with flows of fluid in bidirectional interactions with those new technologies, and particularly applies them to the exploration of vorticity wind turbines (VWT), a new kind of vertical blade-less turbine that gathers energy from the vortex induced vibrations (VIV) of a relatively short and scalable mast. This research presents a framework for such modeling by coupling the discrete element method (DEM) with the Immersed Boundary Method (IBM), for the representation of VWT; and with the finite volume method (FVM), for solving the Navier-Stokes equations. Simulations show that the VWT achieves the lock-in effect for wind velocities between 9 and 15 m/s, with efficiency values between 20 and 30%. The preliminary results together with logistic and cost-related reasons, make these devices very promising, especially when considering the difficulties of implementing new approaches in developing countries.

## 1 Introduction

In recent decades, renewable energies had great expansion worldwide. The causes for this growth are varied and can be associated with several changes, such as:

• A rise in environmental awareness and related policies, as there is currently a strong global pressure to slow down global warming with international commitments to reduce emissions by setting targets and maximum agreed levels.
• An expected depletion of the natural resources currently used for power generation, such as coal and gas.
• The widespread adoption of new generation technologies that are gaining ground in the global market and consolidating their position in the mainstream. That includes not only a growing variety of devices and sources of generation but also the construction of dedicated infrastructure with growing sustainability as a matter of national policy.
• The technological advance of classic renewable generation (traditional wind-turbines and solar panels) that have gained performance efficiency and thus improved their economic equation.
• A growing tendency to diversify the energy matrix in order to avoid dependence on highly fluctuating fossil markets.
• An ever-growing increment in energy demand, related to the development of emergent economies.
• A need for isolated generation in rural areas, away from transmission lines, and even for micro-generation in urban areas, bypassing the construction or overloading of transmission and distribution networks.

Although there have been many substantial technical developments on large-scale wind generation, there are still no technologies with the same positive impact on the economic equation at the micro-generation level, either because of the long pay-back periods that small wind turbines involve or due to the susceptibility of these to the turbulence. So, it is unusual to find this type of installations either in urban or rural environments, as small scale wind infrastructures are still less convenient than micro-generation installations of solar power.

The present research describes and analyzes numerically the generation of electric energy by transforming the kinetic energy of an oscillating structure, induced by the vortex shedding as it is immersed in a fluid flow, commonly known as VIV (Vortex Induced Vibrations). The structure to be analyzed is an inverted vertical cone coupled at the bottom to an elastic bar to which it is anchored, while it leaves the other end free to oscillate. The alternating detachment of the vortices over each side of the cone generates forces in a direction perpendicular to that of the stream flow, with equal frequency as the vortex shedding. These forces generate an angular oscillation of the whole structure, with respect to the anchor point of the elastic rod, which is maximized when the vortex detachment comes into resonance with the natural frequency of the structure.

The generation of electrical power through VIV systems has been the focus of many research studies as it has proven to be a very promising technology, Bernitsas et al., 2008, Bernitsas et al., 2009; Xiao and Zhu (2014); Cajas et al. (2016); Soti et al. (2017); Yáñez Villarreal (2018). In this scenario, it could certainly be very useful to have a tool capable of simulating the operation of these devices in order to contribute to their design and optimization by measuring their power output, resistance, durability, etc. Having the chance to calibrate numerically many design properties in advance, prior to the prototyping stage, could save a lot of time and resources.

On the other hand, the recent increase in the processing power of computers and the development of parallel computation have allowed to devise numerical models suitable for the simulation and resolution of flows in diverse conditions. In particular computational fluid dynamics (CFD) codes played a crucial role, being able to explicitly solve the flow around obstacles and allowing to capture the state of a certain specific situation. Simulations of fluid flows are widely addressed with the finite volume method (FVM), Ferziger and Peric (2002). The discrete element method (DEM) is well suited to represent both loosely elastic structures and flexible bodies (Sassi et al. (2017)). Finally, the adoption of methods like the immersed boundary method (IBM) bring geometrical flexibility, facilitating the study of complex geometries.

Enclosed in this work there is a coupled model where the DEM method was implemented to represent a flexible rod, the IBM was used to represent a rigid body, while the fluid flow was solved using an open-source fully implicit 3D incompressible parallel finite volume flow solver for Navier-Stokes equations in complex geometry, caffa3d.MBRi, presented by Usera et al. (2008).

## 2 Literature review

### 2.1 Vortex induced vibrations

There are many research publications aimed to numerically predict the motion of structural VIV, most of them studying circular cylinders immersed in fluid flows. It is well known that the flow around a fixed circular cylinder can be affected by a number of parameters, including Reynolds number, surface roughness, free stream turbulence level, etc. Likewise, when a dimensional analysis is applied to elastically mounted cylinders there are also important variables to consider (listed in Table 1) related to their structural mass (*m*), structural damping (*c*) and stiffness (*k*= spring constant). These parameters are the most widely explored in the literature, but they are not the only ones that can be considered.

**Table 1:** Non-dimensional groups for a elastically mounted cylinder. Here *m*= total structural mass, *ρ*_*f*_= fluid density, *D*= cylinder diameter; *L*= cylinder length; *ρ*_*m*_= cylinder density; *c*= structural damping; *k*= spring constant. The ideal added mass, *m*_*A*_, is given by *m*_*A*_ = *C*_*A*_ ⋅ *m*_*d*_, where *m*_*d*_ is the displaced fluid mass and *C*_*A*_ is the added mass coefficient. (*C*_*A*_ = 1.0 for a cylinder). *U*= free-stream velocity; *A*= transverse displacement amplitude. The frequency ratio *f*^⁎^ is defined as $\left(f/f_{N_{o}}\right)$, where *f* is the oscillation frequency, and *f*_*N*_ is the natural frequency in the presence of fluid. *F*_*y*_= transverse fluid force; *μ*= fluid viscosity and *f*_*v*_= vortex shedding frequency.

Mass ratio	*m*^⁎^	$\frac{m}{\pi \cdot \rho_{f}\cdot D^{2}\cdot \frac{L}{4}}=\frac{\rho_{m}}{\rho_{f}}$
Damping ratio	*ξ*	$\frac{c}{2\sqrt{k\cdot (m+m_{A})}}$
Mass-damping parameter	*α*	(*m*^⁎^ + *C*_*A*_)⋅*ξ*
Velocity ratio	*U*^⁎^	$\frac{U}{f_{N}\cdot D}$
Amplitude ratio	*A*^⁎^	$\frac{A}{D}$
Frequency ratio	*f*^⁎^	$\frac{f}{f_{N}}$
Transverse force coefficient	*C*_*y*_	$\frac{F_{y}}{\frac{1}{2}\cdot \rho_{f}\cdot U^{2}\cdot D\cdot L}$
Reynolds number	*Re*	$\frac{\rho_{f}\cdot U\cdot D}{\mu }$
Strouhal number	*St*	$\frac{f_{v}\cdot \;L}{U}$

One of the first publications that presented considerable insight about VIV response of a cylinder was written by Parkinson (1972). He considered a mass, spring and dash-pot system driven by the fluid force resulting from vortex shedding. Equation (1) is the differential equation for transverse displacement (*y*) of a bluff body:

(1) $$m\overset{¨}{y}+c\overset{˙}{y}+ky=F_{y}=C_{y}\frac{1}{2}\rho_{f}U^{2}D$$

where the fluid force in the transverse direction, $F_{y}$, is expressed in the second equality; $C_{y}$ is the lift coefficient (tabulated for different geometries); and the dot symbol stands for differentiation with respect to physical time *t*. Parkinson made two important assumptions in his research, the first being that the force and response are sinusoidal with the same frequency (*f*) and secondly that the fluid force leads the response by a constant phase angle Φ, this is the so-called lock-in effect,

(2) $$F_{y}(t)=F_{y_{o}}\sin(\omega t+\Phi )$$

(3) y(t)=Asin⁡(ωt)

where ω=2πf. With these considerations and applying it to a flexible cylinder he obtained relations for the vibration amplitude, Eq. (4), and frequency, Eq. (5), as functions of the previous parameters, where the importance of the phase angle and the role played by mass and damping (mass-damping parameter, *α*) was shown very clearly. *α* has historically been used for the characterization of the flow-induced vibration of cantilevered, flexible structures in wind and it is also known as the Scruton number *Sc*.

(4) $$A^{⁎}=\frac{1}{4\pi^{3}}\;\frac{C_{y}\cdot \sin(\Phi )}{(m^{⁎}+C_{A})\cdot \xi }\;{\left(\frac{U^{⁎}}{f^{⁎}}\right)}^{2}\cdot f^{⁎}$$

(5) $$f^{⁎}=\sqrt{\frac{\left(m^{⁎}+C_{A}\right)}{\left(m^{⁎}+C_{EA}\right)}}$$

Here $C_{A}$ is the flow added mass coefficient and $C_{EA}$ is an “effective” added mass coefficient that includes an apparent effect due to the total transverse fluid force in phase with the body acceleration.

Since Parkinson's research, several other studies have emerged that analyze changes induced in the response of a cylinder under VIV by varying the parameters of Eqs. (2) to (5), as can be seen in Griffin et al. (1975). In the latter, the so-called “Griffin plot” is introduced, where the maximum VIV amplitude was plotted against the Shop-Griffin parameter, $S_{G}=2\pi^{3}\;St^{2}\;(m^{⁎}\xi )$, and it was believed for some time that the amplitude of the VIV was insensitive to Reynolds number changes. However, Klamo et al. (2005) and Govardhan and Williamson (2006) have both demonstrated the strong influence of the Reynolds number on the maximum VIV amplitude of a cylinder. Refer to Williamson and Govardhan (2008); Bearman (2011) for more detailed reviews.

### 2.2 Harvesting energy from VIV

Traditionally, the study of VIV has focused primarily on trying to avoid such synchronization regimes, for example when analyzing its effect on buildings, bridges, underwater structures, etc., Griffin et al. (1975). However, the objective of this study is to examine the problem in the opposite way, that is, to promote the vortex-induced vibrations of a bluff body in fluid flows, with the aim of producing energy with them. In this sense, the oscillation of the body can be used to periodically displace a magnet within a coil, and thus generate electrical energy. The magnet-coil system will induce an electromotive force (emf) on the body, which from the body's perspective can be interpreted as damping. In fact, a damping term is introduced into the body's equation of motion. The rationale behind this idea is to harvest the energy dissipated by this damping system, which results physically feasible, as has already been investigated.

There are already many works aimed at the conversion of hydro-kinetic energy into electrical energy by fostering the VIV of cylinders, Meliga et al. (2011). Bernitsas et al., 2008, Bernitsas et al., 2009 introduce the Vortex Induced Vibrations Aquatic Clean Energy (VIVACE) converter. The research presents the experimental results from a laboratory rig where an integrated power efficiency of $\eta_{vivace}=0.22$ and a peak of $\eta_{peak}=0.31$ was reached.

Barrero-Gil et al. (2012) made a parametric study in the mass ratio ($m^{⁎}$) and the mechanical damping (*ξ*) parameters, to quantify the energy conversion. The efficiency *η* is defined by the ratio of the mean power imparted by the flow to the body per unit length $P_{F-B}$ and the total power resulting in the flow per unit length $P_{F}$, where the total power in the flow per unit length is $\rho U^{3}D/2$; and integrating in one cycle of oscillation we have:

(6) $$\eta =\frac{P_{F-B}}{P_{F}}=\frac{\frac{1}{T}\;\int_{0}^{T}F_{y}\;\overset{˙}{y}\;dt}{\rho U^{3}D/2}$$

Considering a steady state of sinusoidal oscillations with amplitude “*A*” and frequency “*f*” it follows from Eq. (4) and Eq. (6) an expression to the conversion factor, given by Eq. (7), and taking experimental data from forced vibrations tests, they conclude that: (*i*) the efficiency is mainly influenced by the mass-damping parameter and there is an optimum value that maximizes the efficiency; (ii) the range of reduced velocities with significant efficiency is mainly governed by $m^{⁎}$; and (iii) high efficiency values can be achieved for high Reynolds number, η=0.24 was achieved for $m^{⁎}\xi =0.35$ at Re=10,000.

(7) $$\eta =\pi A^{⁎}C_{Y}\sin\Phi \cdot (\frac{f^{⁎}}{U^{⁎}})$$

In Eq. (7), $U^{⁎}/f^{⁎}$ is the true reduced velocity $V^{⁎}$. It should be noted that the efficiency can also be defined considering the total area covered by the oscillation device during its motion. In that case, $\overset{¯}{\eta }=P_{F-B}/P_{F^{⁎}}$, where, in the case of an elastically mounted cylinder, $P_{F^{⁎}}=\rho U^{3}(2A+D)/2$.

In a recent publication, Soti et al. (2017) explores numerically the generation of electrical power from VIV on a cylinder. In their work, the cylinder is free to oscillate in the transverse direction, attached to a magnet that can move along the axis of a coil made from conducting wire. For the calculation of the electromagnetic force they use the single magnetic dipole approximation proposed by Donoso et al. (2010) that includes a mathematical model of the interaction between a N−turns coil and a single oscillating magnet. The magnetic force can be considered as a damping force with a non-constant damping coefficient and the electrical power is calculated by multiplying the electromagnetic force with the velocity of the magnet.

### 2.3 Computational fluid dynamics, CFD

Since the late 1990s, numerous open source codes have been developed and made available to solve the Navier-Stokes equations, Zaleski (2001). Among them, different grid topologies can be found, ranging from fully structured grids, either orthogonal or not Lehnhäuser and Schäfer (2003), through block-structured grids Lilek et al. (1997), and up to fully unstructured ones. In terms of geometric capacity, methods such as local grid refinement (Lange et al. (2002)) or the immersed boundary (Liao et al. (2010); Mendina et al. (2014)) have facilitated the computational study of complex bodies in interaction with fluids.

In this work we present a coupled model where a simple structural DEM method is implemented to represent the elastic rod, which is suitable to represent freely moving bodies and elastic structures. The fluid flow is solved using caffa3d.MBRi, an open-source fully implicit 3D incompressible finite volume solver for Navier-Stokes equations in complex geometry. Please refer to Usera et al. (2008); Mendina et al. (2014) for a full description, validation and mesh and time independence studies of the flow solver.

#### 2.3.1 Immersed boundary method, IBM

The immersed boundary method is a natural alternative to body-fitted or unstructured-grid methods when the aim is to simulate flow in domains with complex rigid boundaries, such as in fluid-solid interaction problems, which generally results in larger requirements of memory and involves higher computational costs. The IBM method not only has the advantage of keeping the structured Cartesian grids but also provides the possibility to handle more complex geometries. The method was first implemented by Peskin (1972), and then generalized in Peskin (2002), involving the add of a source term (or forcing function, $f_{b}$) in the governing equations to reproduce the effect of the boundary and where two main approaches are found, direct and feed-back forcing.

In the present work we have used the immersed boundary method, which was already incorporated in caffa3d.MBRi. Refer to Mendina et al. (2014) for research that validates the method. Here it is used to model the mast of the VWT (see Section 3.2), represented as an inverted cone, using the direct forcing approach, proposed by Mohd-Yusof (1998). In this approach the governing equations are discretized in the Cartesian grid. Then a forcing term (body force) is added to the source term that corresponds to the cells inside the immersed boundary. This ensures an easier control over numerical accuracy, stability and conservation properties of the solver, so as to ensure that the desired velocity distribution at the boundary is satisfied. This approach was further extended by Fadlun et al. (2000), refer to Mittal and Iaccarino, 2005a, Mittal and Iaccarino, 2005b for a deeper review and examples of the different kinds of approaches.

#### 2.3.2 Discrete element method, DEM

The DEM is a numerical method used for many kinds of applications. It is especially advantageous for calculating the dynamics of a large number of small particles. The proper use of the method requires a correct interpretation of the system to be represented, as a set of objects, whose interactions are governed by known laws. In this way, significant results can be obtained, that is, consistent with reality. The theory and elements to consider in particle simulations with the DEM are described in Hockney and Eastwood (1988).

In the field of rocks mechanics, a discontinuous analysis was first used by Cundall and Strack (1979). The method consists of deformable blocks and deformable contacts that become frictional contacts after failure. Today DEM has become widely accepted as an effective method for addressing engineering problems in granular and discontinuous materials, especially in granular flows, powder mechanics, and rock mechanics. Nowadays its applications are being extended to analyze load and deformation on flexible and elastic systems in addition to the initial application for determining the failure of structures.

In extensive reviews of the method, Zhu et al., 2007, Zhu et al., 2008, several studies were reported in which the DEM has been coupled with CFD models to simulate particle-fluid flows. In most of the literature, millions of particles without significantly positional bonds are considered. However, other authors used the method to represent elastic systems of a relatively small number of particles interconnected by ideal springs. In the latter approach, the elastic bonds given by the springs represent the main constraint applied to the particles. The overall shape and stress of the system is then estimated by calculating the displacements of these point masses (particles). This type of approach was used in a previous work Sassi et al. (2017).

In Ivanov (2001) a three dimensional DEM program is developed, for the analysis of engineering structures subject to earthquake loads. A chapter of his Ph.D. thesis is dedicated to the analysis of shell structures with the DEM. In his work, he represents the in-plane stiffness of shells by a lattice of energy equivalent to normal springs, while the bending stiffness of walls is represented by bending springs. He considers the elements of the lattice as beams that can transfer normal $F^{n}$, shear $F^{s}$ and bending forces *M*. The method consists on a double integration of the Newton equations of motion of each element, as the motion of an element is considered uncoupled from the motion of the rest of the elements during a time step, there is no need to assemble a stiffness matrix. Instead, the coupling is obtained by updating the spring forces at every time step; each element will move, in the next step, due to the unbalance on the summation of forces in all springs attached to the element. Two types of damping are applied to reach equilibrium: the relative motion between elements of the structure is damped by a coefficient of damping calculated from the critical damping ratio of the material. Secondly, due to the motion of the structure immersed in a fluid, there is a small amount of viscous damping, that represents the resistance of the medium in which the structure is placed. In Section 3.3 Ivanov's algorithm will be adjusted to represent the dynamics of the flexible rod of the Vorticity Wind Turbine, VWT.

## 3 Modeling

The VWT structure consists of four essential parts:

• **Foundation:** the underground structure that adds stability to the turbine.
• **Generation system:** that transforms the kinetic energy from the oscillations into electricity by a built-in linear alternator.
• **Rod:** made of carbon fiber, it provides strength and flexibility to the movement, while minimizing energy dissipation and providing the highest resistance to fatigue. It penetrates into the mast for 20 percent of the mast length, it's anchored to it at its top end and the bottom is secured to the foundation.
• **Mast:** a light conic structure, built using resins reinforced with carbon and glass fibers, designed to be rigid and remaining anchored to the rod, at its bottom, and free on the top to guarantee the maximum amplitude of the oscillation.

### 3.1 Computational domain

The strategy to analyze the VWT structure is to divide it in two main parts, the rod and the mast, and treat the forces acting over these parts separately. The rod is analyzed as a beam anchored vertically to the ground with the DEM method, while the mast is represented by the Immersed Boundary Method.

**Figure 1:**
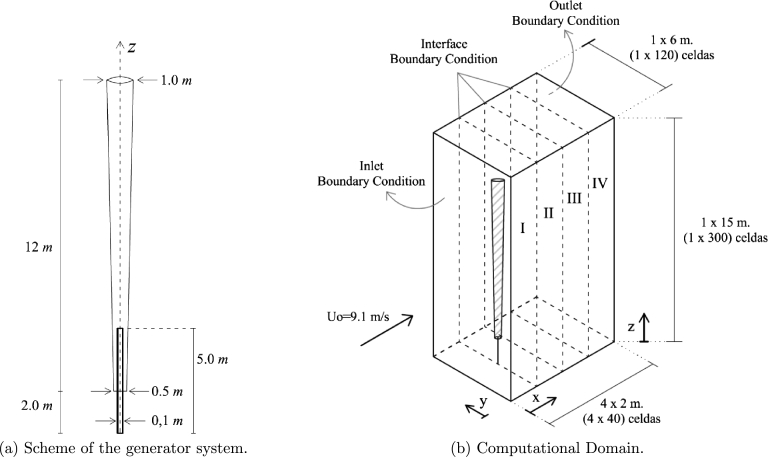
Scheme of the Vorticity Wind Turbine and computational domain.

The domain of the simulations is an (8×6×15) meters prism in the x,y and *z* directions respectively. It is divided in cubic cells of 5cm side distributed in four blocks and regions of (2×6×15) meters, one block for each region, named as blocks I to IV from west to east (*x* direction), see Fig. 1b. Block I, has inlet boundary condition on its west face, where a uniform flow ($U_{o}=9.1\;m/s$) in the *x* direction enters the domain. The in-between faces, represented by the dashed lines, have interface boundary conditions. Block IV, has null-gradient outlet boundary condition on its east boundary. Finally, all the north, south, top and bottom boundaries of the four blocks have non-slip wall boundary conditions.

One processor is used for each region, using Message Passing Interface (MPI). The whole domain consists of (160×120×300) cells adding a total of 5,760,000 cells, evenly distributed across the 4 processors (1.44 millions each). The time step for the fluid flow solver is dt=0.01sec. The wall time for computing 1 second of simulation is almost one hour.

While a tight domain size was selected in sake of computational efficiency, no artifacts were detected on the flow that could have been induced by outlet or lateral boundary conditions. The simplified condition of uniform flow at the inlet will be addressed in a future work, incorporating specific boundary layer profile and turbulence intensity levels at inlet.

### 3.2 Mast

The mast is an inverted cone as shown in Fig. 1a, represented as a rigid structure with the direct forcing approach of the IBM. It is represented mathematically at each time step as a function of the position and normal direction of the last element of the rod (described in Section 3.3).

The source term corresponding to the cells inside of the mast and its boundary is modified to make the velocity of each cell equal to the translational velocity of the mast. This can be interpreted as a mass field $\overset{\to }{F^{⁎}}$ that acts on the inner cells, and thus, the force exerted by the mast over the fluid is calculated by integrating the mass field in a region (*D*) enclosing the mast:

(8) $$F_{imb}=-\int_{D}\rho \;\overset{\to }{F^{⁎}}\;dV$$

the same can be applied to calculate the moment that the overall force exerts over the connecting node (*CN*) of the elastic rod, the highest node of the rod, where the mast and the rod are connected.

(9) $$M_{imb}=-\int_{D}(P-O)\wedge (\rho \;\overset{\to }{F^{⁎}})\;dV$$

Where (P−O) is the distance from every computing point from the region (*D*) to the connecting node. Once the force that the fluid exerts over the mast ($F_{imb}$) and the moment with respect to the connecting node ($M_{imb}$) are calculated, the procedure continues with the analysis of the rod.

### 3.3 Rod

**Figure 2:**
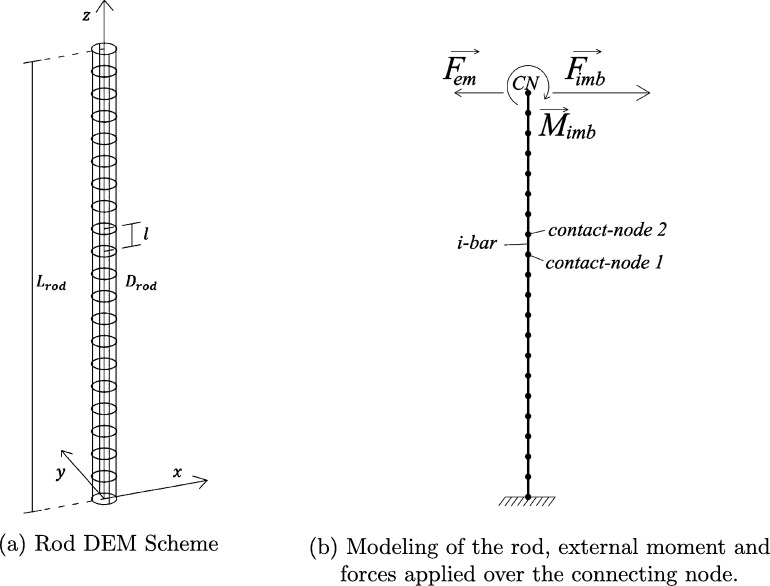
Schematic of the modeling of the Rod with the DEM.

**Figure 3:**
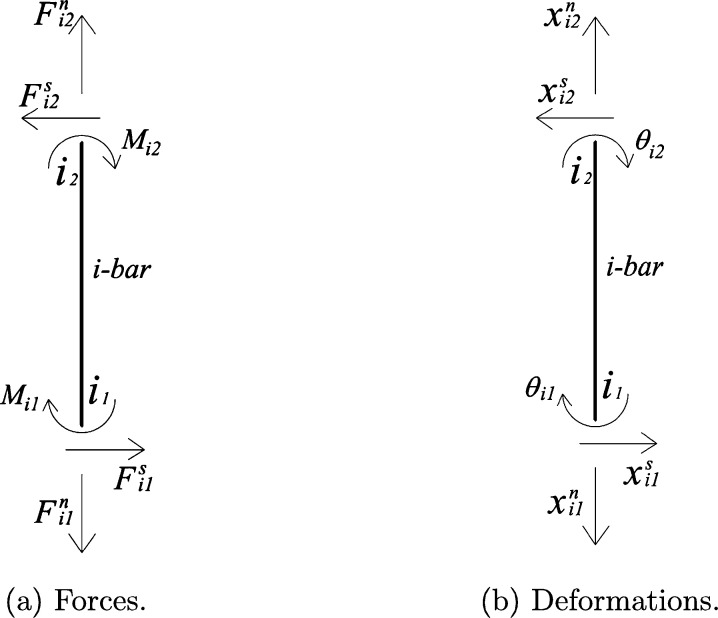
Scheme of the forces applied to a single bar and their corresponding deformations.

The rod is the main section for the modeling of the structure because of its flexibility and elasticity. It is constructed with the DEM approach proposed by Ivanov (2001), composed of 20 equal bars of length l=10cm and diameter $D_{rod}=10\;cm$, that conform the 2 meters rod ($L_{rod}$), see Fig. 2a. Note that, as the mast is modeled with IBM as a rigid body, there is no bending taking place in the part of the rod that penetrates into the mast, and for that reason only the first two meters of the rod has to be modeled. Each bar is constructed as a linear bar with two punctual nodes, sharing with the next bar one of those punctual nodes at each end, schematic representation in Fig. 2b. The nodes are the contact points in which the forces and moments are transmitted from bar to bar. Fig. 3 shows the schematic representation of forces in Fig. 3a and the corresponding deformations in Fig. 3b, the bar is named with a counter “*i*” (from 1 to 20) and the nodes are numbered for each bar “$i_{1}$” and “$i_{2}$”. The external forces, and moment are applied in the highest node, named as “Connecting-Node” (CN) because it is the one that connects the rod and the mast.

#### 3.3.1 Update of velocities

The motion of each element of the rod is represented by the first and second cardinal equations, applied at the center of the bars and used to calculate the displacement and rotation of the contact nodes (endings). The first, Eq. (10), is the equation for translational motion, and the second for rotational motion, Eq. (11), of a single bar “*i*”.

(10) $${\overset{¨}{x}}_{i}+\alpha \;{\overset{˙}{x}}_{i}=\frac{F_{i}}{m_{i}}+g$$

(11) $$\overset{˙}{\omega_{i}}+\alpha \;\omega_{i}=\frac{M_{i}}{I_{i}}$$

where $x_{i}=$ position vector; $\omega_{i}=$ rotational velocity; $m_{i}=$ mass; $I_{i}=$ mass moment of inertia; and *g*= gravity acceleration. The dots represent the derivative in time. A centered finite difference is used for the integration of the equations of motion. New and old values, within a time step, are designated by superscripts plus and minus respectively, thus the expression for translational and rotational velocities are provided by

(12) $${\overset{˙}{x}}_{i}=\frac{1}{2}\;[{\overset{˙}{x}}_{i}^{-}+{\overset{˙}{x}}_{i}^{+}]$$

(13) $$\omega_{i}=\frac{1}{2}\;[\omega_{i}^{-}+\omega_{i}^{+}]$$

and the expression for translational and rotational accelerations,

(14) $${\overset{¨}{x}}_{i}=\frac{1}{\Delta t_{dem}}\;[{\overset{˙}{x}}_{i}^{+}-{\overset{˙}{x}}_{i}^{-}]$$

(15) $${\overset{˙}{\omega }}_{i}=\frac{1}{\Delta t_{dem}}\;[\omega_{i}^{+}-\omega_{i}^{-}]$$

inserting this expressions in the equations of motion, Eq. (10) and Eq. (11), and solving for new values of velocities results in:

(16) $${\overset{˙}{x}}_{i}^{+}=[D_{1}\;{\overset{˙}{x}}_{i}^{-}+(\frac{F_{i}}{m_{i}}+g)\Delta t_{dem}]D_{2}$$

(17) $$\omega_{i}^{+}=[D_{1}\;\omega_{i}^{-}+(\frac{M_{i}}{I_{i}})\Delta t_{dem}]D_{2}$$

where $D_{1}=1-(\alpha \;\Delta t_{dem}/2)$, and $D_{2}=1/[1+(\alpha \;\Delta t_{dem}/2)]$, with $\Delta t_{dem}=$ time step for the DEM. The time step used for the displacement of the rod is smaller than the time step used by the fluid solver (caffa3d.MBRi), as the differential equations system governing the motion of the rod is too stiff, because of the high Young's module of the material, and then it is necessary to use a smaller time step than the one required for solving the fluid. In the present work, the ratio between the fluid solver and DEM time steps is 10,000, this means that the DEM runs 10,000 times for each fluid solver time step. Anyway, as the number of nodes representing the rod is much smaller than the number of fluid cells $(2\times 10^{1}$ vs $5\times 10^{6})$ this difference between the time steps does not introduce significant inefficiencies in the overall time of the simulation, and the convergence of the system is guaranteed.

#### 3.3.2 Update of contact forces

**Figure 4:**
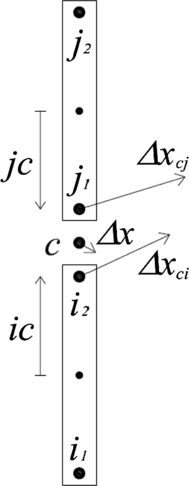
Scheme of the contact node.

With the new velocities obtained, it is possible to update the contact forces in the nodes in between bars, which depend on the properties of the system. In the following description, the contact between two consecutive bars *i* and *j* is considered, as outlined in Fig. 4. To update the normal and shear forces, and bending moment, first the incremental displacements of the contact point due to translational and rotational motions of the elements are computed using the new values of velocities. Note that for each bar there are two nodes (one at each end) denoted as ‘$i_{1}$’ and ‘$i_{2}$’ for bar *i*, and $j_{1}$ and $j_{2}$ for bar *j*. As the contact node $c=i_{2}=j_{1}$ belongs to both bars, it has two displacements. One obtained by computing on bar *i*, $\Delta x_{ci}$ and the other by computing on bar *j*, $\Delta x_{cj}$. Eq. (18a) calculates the translational displacement for the contact node obtained by computing on both bars.

(18a) $$\Delta x_{ci}^{tr}={\overset{˙}{x}}_{i}^{+}\Delta t\;\Delta x_{cj}^{tr}={\overset{˙}{x}}_{j}^{+}\Delta t$$

(18b) $$\Delta x_{ci}^{rot}=ic^{+}\times \Delta \theta_{i}\;\Delta x_{cj}^{rot}=-jc^{+}\times \Delta \theta_{j}$$

Where $ic^{+}$ and $jc^{+}$ are the current vectors from the center of the bar to the contact points, and $\Delta \theta_{i}$ and $\Delta \theta_{j}$ are the incremental rotations of the bars. For the first time step the method uses values of position and velocity provided as initial conditions. Thus, the total incremental displacement at the contact is:

(19) $$\Delta x_{c}=(\Delta x_{cj}^{tr}-\Delta x_{ci}^{tr})+(\Delta x_{ci}^{rot}-\Delta x_{cj}^{rot})$$

which resolves into normal and shear components as:

(20) $$\Delta x_{c}^{n}=(\Delta x_{c}\cdot n_{ij}^{+}\;\prime )\;n_{ij}^{+}$$

(21) $$\Delta x_{c}^{s}=\Delta x_{c}-\Delta x_{c}^{n}$$

here $n_{ij}$ is the unit normal vector pointing from bar “*i*” to bar “*j*”; the supra-index “′” stands for the transposed vector (so as to be able to calculate the product). And the angular displacements for bars “*i*” and “*j*” can be calculated as:

(22) $$\Delta \theta_{i}=\omega_{i}^{+}\cdot \Delta \;t,\;\Delta \theta_{j}=\omega_{j}^{+}\cdot \Delta \;t,$$

then, the increment of force in the normal spring is expressed in Eq. (23), where $A_{t}=$ transversal area; *E*= Young's modulus; *l*= element length; and *ν*= Poisson's ratio (material property).

(23) $$\Delta F_{c}^{n}=k_{n}\;\Delta x_{c}^{n},\;\text{with}\;k_{n}=\frac{\sqrt{3}}{3}\;\frac{A_{t}\;E}{l\cdot (1-\nu )}$$

The calculation of the increments of the shear force and bending moment is not as simple as the normal force and further calculations are needed. The relation between these forces and the corresponding displacement for the endpoint $i_{1}$ of the bar ‘*i*’ with second moment of inertia $I_{i}$ is expressed in Eq. (24) and Eq. (25),

(24) $$F_{i_{1}}^{s}=\frac{12EI_{i}}{l^{3}}(x_{i1}^{s}-x_{i2}^{s})-\frac{6EI_{i}}{l^{2}}(\theta_{i1}-\theta_{i2})$$

(25) $$M_{i1}=\frac{6EI_{i}}{l^{2}}(x_{i1}^{s}-x_{i2}^{s})-\frac{4EI_{i}}{l}(\theta_{i1}-\frac{\theta_{i2}}{2})$$

with symmetric relations for node $i_{2}$, where *x*= position vector and *θ*= rotation of the element, see Fig. 3b. Following an analogous course of thought in defining $k_{n}$ in the normal force, an additional set of spring constants can be defined accounting for the bending behavior equal to the coefficients of Eq. (24) and Eq. (25) as follows: $k_{qq}=12EI/l^{3}$ (stiffness of a shear spring for a beam element), $k_{qm}=k_{mq}=6EI/l^{2}$ (stiffness of a shear-rotational spring for a beam element) and $k_{mm}=4EI/l$ (stiffness of a rotational spring for a beam element). Then the respective contributions to the driving force and driving moment are calculated as functions of the relative incremental shear displacement $\Delta x_{c}^{s}$, and the incremental rotations of the elements $\Delta \theta_{i1}$ and $\Delta \theta_{i2}$.

(26) $$\Delta F_{i1}^{s}=-\Delta F_{i2}^{s}=k_{qq}\Delta x_{c}^{s}+k_{qm}\cdot n_{ij}^{+}\times (\Delta \theta_{i1}+\Delta \theta_{i2})$$

Before increasing the values of the contact forces, their old directions have to be corrected in order to reflect the latest orientation of the contact plane. The old normal force should be modified to be collinear to the latest unit normal vector.

(27) $$F_{c}^{n-}=(F_{c}^{n-}\cdot n_{ij}^{+}\;\prime )\cdot n_{ij}^{+}$$

The shear contact force is first updated to be co-planar with the latest contact plane,

(28) $$F_{c}^{s-}=a^{+}\cdot (a^{-}\cdot F_{c}^{s-}\;\prime )+b\cdot (b\cdot F_{c}^{s-}\;\prime )$$

where $a^{+}$, $a^{-}$ and *b* are auxiliary unit vectors given by the expressions $b=(n_{ij}^{-}\times n_{ij}^{+})/|(n_{ij}^{-}\times n_{ij}^{+})|$; $a^{-}=b\times n_{ij}^{-}$ and $a^{+}=b\times n_{ij}^{+}$. Finally, the rotation of the contact shear force has to be considered along the $n_{ij}$ axis. The average rotation along this axis will be $\Delta \theta =1/2\cdot (\Delta \theta_{i1}+\Delta \theta_{i2})\cdot n_{ij}^{+}\;\prime$. The final updated old shear contact force (co-planar with the new contact plane) will be,

(29) $$F_{c}^{s-}=\cos\Delta \theta \cdot F_{c}^{s-}+\sin\Delta \theta \cdot (a^{+}\times F_{c}^{s-})$$

once having corrected the direction of the old contact forces, the incremented ones will yield the new values, with $\Delta F_{i1}^{n}=-\Delta F_{i2}^{n}$ and $\Delta F_{i1}^{s}=-\Delta F_{i2}^{s}$,

(30a) $$F_{i1}^{n+}=F_{i1}^{n-}+\Delta F_{i1}^{n}\;F_{i2}^{n+}=F_{i2}^{n-}+\Delta F_{i2}^{n}$$

(30b) $$F_{i1}^{s+}=F_{i1}^{s-}+\Delta F_{i1}^{s}\;F_{i2}^{s+}=F_{i2}^{s-}+\Delta F_{i2}^{s}$$

Resuming Eq. (25), the increment of moments is computed as:

(31) $$\Delta M_{i1}=k_{qm}\;n_{ij}^{+}\times \Delta x^{s}-k_{mm}\;(\Delta \theta_{i1}+\Delta \theta_{i2}/2)$$

(32) $$\Delta M_{i2}=k_{qm}\;n_{ij}^{+}\times \Delta x^{s}-k_{mm}\;(\Delta \theta_{i2}+\Delta \theta_{i1}/2)$$

and the increment of the moment at both ends:

(33) $$M_{i1}^{+}=M_{i1}^{-}+\Delta M_{i1},\;M_{i2}^{+}=M_{i2}^{-}+\Delta M_{i2}$$

#### 3.3.3 Compute external forces

This iterative procedure is computed for every bar composing the rod. When computing on the highest bar, the force that the fluid exerts over the mast ($F_{imb}$) is transmitted to the connecting node (the one that is connected to the mast), as well as the moment with respect to this node ($M_{imb}$), calculated in the previous Section 3.2 with the IBM. It should be also considered at this stage the magnetic force that the generator exerts over the rod ($F_{em}$), as it is also applied to the connecting node, see Fig. 2b. In the next Section 3.4, it will be explained how that magnetic force should be calculated.

(34) $$F_{CN}^{+}=F_{CN}^{-}+\Delta F_{CN}+F_{imb}+F_{em}$$

(35) $$M_{CN}^{+}=M_{CN}^{-}+\Delta M_{CN}+M_{imb}$$

Having computed the stresses and moment resultants acting on each element, the system is ready to enter a new time step. It is worth highlighting that as the time step for this procedure is smaller than the one for solving the fluid equations, the same immersed boundary force and moment are used during several iterations until the fluid solver time step is advanced.

### 3.4 Linear generator

**Figure 5:**
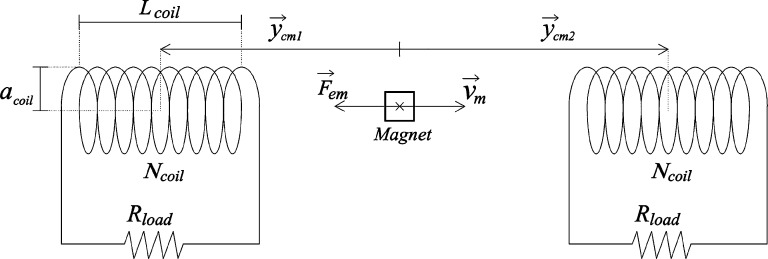
Scheme of the generator system.

The kinetic energy of the oscillations is converted into electricity using a linear built-in generator as the one described by Donoso et al. (2010). It is composed of a magnet coupled to the last bar of the rod, modeled as a punctual magnet, and two fixed coils, one at each side of the rod, see Fig. 5. When the system is oscillating, the magnet oscillates with the rod, generating an oscillating magnetic field inside the coils. According to Faraday's law of electromagnetic induction, the motion of the magnet produces emf (*ϵ*) across the coil. Connecting the coil to a resistive load, an induced current appears, opposing the motion of the magnet by applying an electromagnetic force ($F_{em}$), which can be considered as a damping force in the motion of the rod.

The magnetic force that each coil exerts over the magnet is a function of the magnet velocity and its relative position to the coils, as well as to the properties of the coils and the magnet, and the resistive load connected to each circuit. Here two identical coils are disposed at each side of the rod, connected to two identical loads for reaching a symmetrical displacement. In equilibrium conditions the centers of the coils are located at 8.5 cm in *y*− direction at each side of the magnet. The properties of the magnet and the coils are listed in Table 2.

**Table 2:** Parameters used in the model of the generator.

Property	Value	Unit
*L*_*coil*_	7.0	*cm*
*a*_*coil*_	5.0	*cm*
*N*_*coil*_	700	turns
*R*_*load*_	1 - 100	Ω
*μ*_*m*_	4.5e-3	*J*/*T*
$y_{cm,1}^{0}$	−8.5	*cm*
$y_{cm,2}^{0}$	+8.5	*cm*

From the literature, the equation of the magnetic force ($F_{em,i}$) is obtained as follows:

(36) $$F_{em,i}=c_{m0}\cdot g_{i}^{2}\cdot \overset{˙}{y}\;\text{with}\;c_{m0}=\frac{\mu_{m}^{2}}{R}$$

where $\mu_{m}$ is the magnetic moment of the magnet. Here it is constant because the resistive load (*R*) is set to be constant, but it could be variable, for instance, within the wind velocity. $g_{i}(y)$ is a function of the relative position between the *i*-coil and the magnet $\left(y_{cm,i}\right)$, expressed as:

(37) $$g_{i}(y)=\frac{2\pi N_{coil}a_{coil}^{2}}{L_{coil}}\cdot [\frac{1}{{\left(a_{coil}^{2}+{\left(y_{cm,i}-L_{coil}/2\right)}^{2}\right)}^{3/2}}-\frac{1}{{\left(a_{coil}^{2}+{\left(y_{cm,i}+L_{coil}/2\right)}^{2}\right)}^{3/2}}]$$

where the properties of the coil, denoted with the subindex “coil”, are illustrated in Fig. 5, together with the distance from the magnet to each coil, $y_{cm,i}$.

The magnetic forces are calculated at each time step of the DEM, added in a single force ($F_{em}$), and applied to the highest bar of the rod as expressed in Eq. (34). Note that the magnet is also modeled as a punctual element. As this force opposes the motion of the bar and it is proportional to its velocity, it can be interpreted as a damping force.

To calculate the output power, *P*, the electromagnetic force is multiplied by the magnet velocity at each time step, the negative value is because the force opposes the motion,

(38) $$P(t)=-F_{em}\cdot v_{mg}$$

in this way, the output power is determinate just for an instant of time. Integrating the instantaneous power over a period of time $T^{⁎}$, the accumulated energy is obtained, and dividing then by the value of that period of time, the average output power, $\overset{¯}{P}$, is obtained:

(39) $$\bar{P}=\frac{E_{T}}{T^{⁎}}=\frac{1}{T^{⁎}}\cdot \int_{T^{⁎}}P(t)\cdot dt$$

Finally, the efficiency of the VWT is defined as the ratio of the output power to the power available in the fluid flow over the region occupied by the turbine, $P_{W}$. The latter is constant in this simulations because a uniform flow is used. When dividing the instantaneous power, then the instantaneous efficiency is obtained, *η*; and the integrated efficiency, $\overset{¯}{\eta }$, is obtained dividing the integrated power by $P_{W}$:

(40) $$\bar{\eta }=\frac{\bar{P}}{1/2\;\rho \;U^{3}A_{sw}}$$

where *U* is the velocity of the fluid flow upstream, and $A_{sw}$ is the swept area by the oscillation of the VWT in a plane perpendicular to the flow direction.

### 3.5 Validation of the method

The coupling between the DEM and the FVM has been validated in Sassi et al. (2017) for the simulation of fishnets tunnels under fishing load. The representation of the fishnet with the discrete element method is very similar to that used in the elastic rod of the VWT, where the bars represent the ropes and the nodes represent the knots of the fishnet, with the exception that the ropes does not consider bending moment transmission. The results were compared with experimental data from a towing tank.

In the modeling of the elastic rod, the bending moment transmission developed by Ivanov (2001) has been incorporated. The same method was used in Salles et al. (2018) for the numerical modeling of a stringer stiffened silo collapse under eccentric discharge flow. Again, in the latter study, numerical results were contrasted with physical models of collapsing silos.

## 4 Results

The modeling of a vorticity wind turbine (VWT), as explained in the previous section, was carried through and simulated numerically with caffa3d.MBRi. Results are presented in this section, in a first instance, for a uniform air flow of $U_{o}=9.1\cdot m/s$, as this is the critical velocity; this means that the vortex shedding frequency is equal to the natural frequency of the structure. The Reynolds number calculated at the equivalent height $h_{e}=8.4\;m$ (60% of the mast height) for the critical velocity is $Re=4.67\times 10^{5}$. Following that, a sensitivity analysis of wind speed and other relevant parameters will be shown. In all the simulations presented here, a blending coefficient of 0.95 is used together with a simple LES Smagorinsky turbulence closure model.

**Figure 6:**
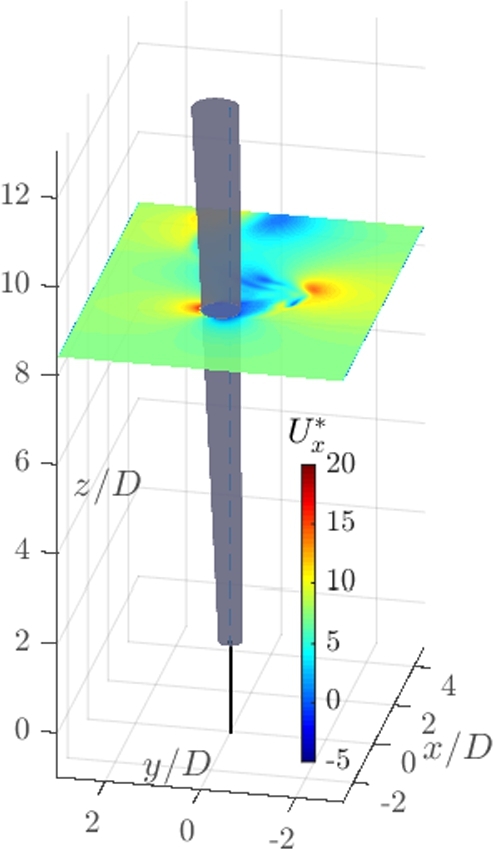
Three-dimensional view of the Simulation with the velocity field displayed in the x-direction over an horizontal plane at a height of 80% of the mast. The associated animation can be viewed in the electronic rendition of this article at: https://doi.org/10.1016/j.heliyon.2020.e05155.

Fig. 6 shows the simulated VWT. The reduced velocity field is displayed in the x−direction over the horizontal plane. The grey cone is the mast, represented by the IBM and the thin black line is the rod, represented by the structural DEM as a linear bar, invisible to the fluid. The vortex detachment can be seen in the velocity field.

**Figure 7:**
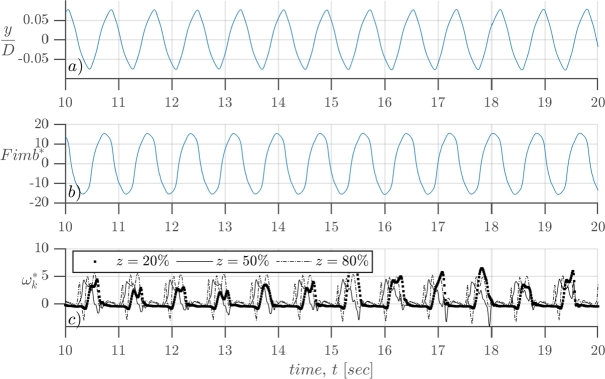
*a*) Dimensionless transverse displacement of the top of the rod, where the magnet is located *b*) dimensionless integrated transverse force, $F_{imb}^{⁎}=F_{imb}/(\rho {\left(UD\right)}^{2})$, and *c*) dimensionless in-plane vorticity calculated at three monitoring points corresponding to 20, 50 and 80 % of the length of the mast.

Fig. 7, *a*) shows the reduced transverse displacement of the top of the rod ($y^{⁎}=y/D$ with *D* the diameter at the top of the mast), where the magnet is located, for 10 seconds of simulation with a uniform air flow of $U_{o}=9.1\;m/s$. The oscillatory response shows that the lock-in effect is well captured by the coupled simulation and that the device is taking energy from the fluid. This is evidenced by the sinusoidal displacement, of amplitude A=0.15D, which correlates with the graph of the transverse displacement for structures under the lock-in effect. Note that Fig. 7 a) plots the displacement of the top of the rod, where the magnet is located. And that it has no dimensions because it has been divided by the diameter of the top of the mast (D=1m). Also, the mast is much longer, and so, the amplitude of the oscillations is much wider. For this same case, the amplitude of the transverse displacement at the top of the mast is twice its diameter.

Panel *b*) of Fig. 7 plots the dimensionless integrated transverse force acting over the mast, obtained by dividing the integrated force by $\rho {\left(UD\right)}^{2}$. Finally, the reduced in-plane vorticity, $\omega_{k}^{⁎}$, defined in Eq. (41), is calculated in three monitoring points at every 0.01 seconds. It is plotted vs time in panel *c*) of Fig. 7. The three monitoring points are set for the same *x* and *y* coordinate components, downstream the turbine, and at three different heights, $z_{1}$, $z_{2}$ and $z_{3}$ (corresponding to 20%, 50% and 80% of the height of the mast respectively). In the discretized domain, the two-dimensional vorticity for the (i,j) node in the *k* direction, is calculated with the least-square method in Eq. (42).

(41) $$\omega_{k}=(\nabla \wedge \overset{\to }{v})\cdot \overset{ˆ}{k}\;\omega_{k}^{⁎}=\frac{\omega_{k}}{U_{o}/D}$$

(42) $$\omega_{k}(i,j)=\frac{2\cdot v_{i+2,j}+v_{i+1,j}-v_{i-1,j}-2\cdot v_{i-2,j}}{10\cdot \Delta x}-\frac{2\cdot u_{i,j+2}+u_{i,j+1}-u_{i,j-1}-2\cdot u_{i,j-2}}{10\cdot \Delta y}$$

**Figure 8:**
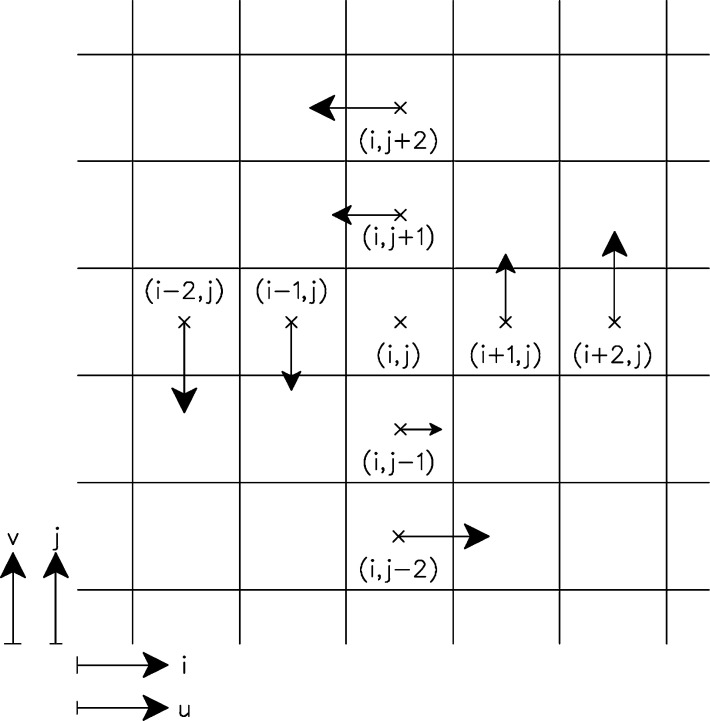
Scheme of the vorticity calculation at the node (*i*,*j*).

Where *i*, *j* and *k* are the cell index of the *x*, *y* and z−coordinates respectively, *u* and *v* are the velocity components in the *x* and y−direction, and Δ*x* and Δ*y* are the distance between two consecutive computing points in *x* and y−direction respectively. In Fig. 8 a scheme of the calculation is shown, note that the situation on the figure is a simplified case where the maximum vorticity is obtained, because every term on the right hand side of Eq. (42) adds a positive value to the final $\omega_{k}$.

The main concept drawn from the plotted data in Fig. 7 panel *c*), is that the shedding at the three vertical positions, $z_{1}$, $z_{2}$ and $z_{3}$ (along the height of the mast) are synchronized. This is evidenced by the simultaneous peaks and valleys of the dotted, continuous and dashed lines. Because of this, the transverse force integrated along the structure, at a given instant of time, will be maximized. Otherwise, opposing forces will coexist along the structure, and the resulting force will be reduced.

**Figure 9:**
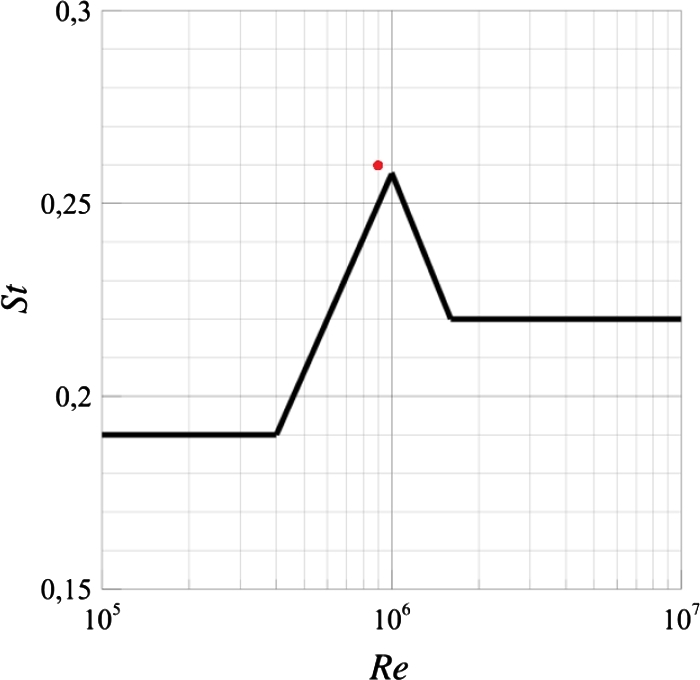
Strouhal number as function of Reynolds number for cylinders, taken from Advisory Committee on Technical Recommendations for Construction (2010), the red dot is the Strouhal number calculated from the simulations.

**Figure 10:**
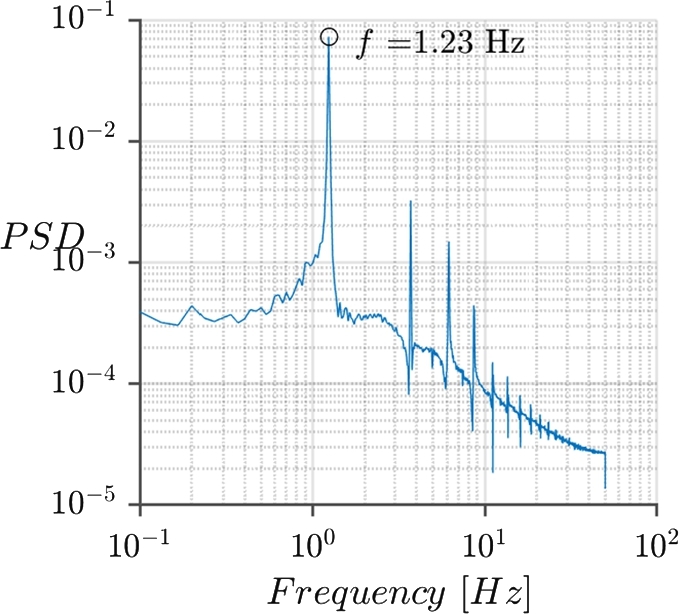
Spectral analysis of the displacement of the top of the rod (magnet location).

Carefully looking at Fig. 7 *a*), slight difference between peaks and valleys can be found in the plot. This indicates that several frequencies are presented in the dynamics. In order to verify the dynamics of the oscillation, the Strouhal number and the Reynolds number at the top of the VWT have been calculated. The red dot in Fig. 9 represents the working point of the simulation, and the black line, taken from Advisory Committee on Technical Recommendations for Construction (2010), is the Strouhal number as a function of the Reynolds number for cylinders. Also, a spectral analysis of the oscillations has been made, Fig. 10, in order to determine the main oscillatory frequency of the turbine, f=1.23Hz.

**Figure 11:**
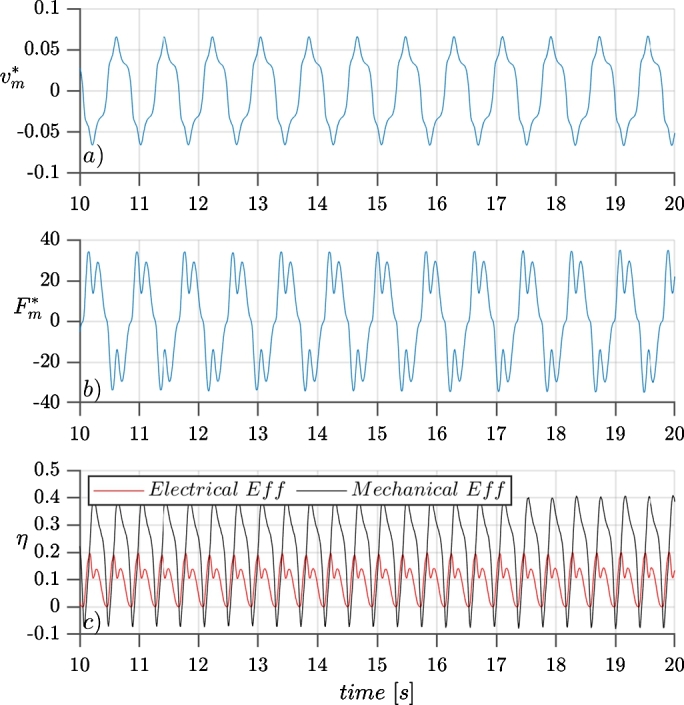
Plot of the magnet velocity (upper panel), magnetic force (mid panel), and instantaneous mechanical and electrical output power (bottom panel), during 10 seconds of simulation with uniform wind velocity of *U*_*o*_ = 9.1 *m*/*s*.

To quantitatively assess the output energy production, the calculations of Section 3.4 were followed. In Fig. 11 three plots are shown: the upper panel (*a*) plots the reduced velocity of the last element of the rod (where the magnet is fixed, and thus this velocity is equal to the magnet velocity) during 10 seconds of the simulation. The mid panel plots the reduced magnetic force calculated at every time step for a generator with the properties of Table 2 connected to a resistive load of R=7Ω. Multiplying these two values (magnet velocity and magnetic force) at every time step, the instant output electrical power is obtained, plotted in the bottom panel (*c*) together with the instant mechanical power available in the last element of the rod, the latter calculated as expressed in Eq. (44). Both powers are reduced with the power available in the wind Eq. (43), therefore, the plotted data correspond to the mechanical and electrical efficiencies of the VWT. The swept area is calculated knowing the amplitude of the angular displacement, in this simulation, with an angular amplitude of 10 degrees, the swept area is $A_{sw}=16.23\;m^{2}$.

(43) $$P_{wind}=1/2\;\rho \;U^{3}\;A_{sw}$$

(44) $$P_{mec}=M_{imb,x}\cdot \omega_{x}+F_{imb,y}\cdot v_{y}\;\eta_{mec}=\frac{P_{mec}}{P_{wind}}$$

(45) $$\eta^{⁎}=\frac{P_{e}}{P_{wind}}$$

Here a peak efficiency of $\eta_{peak}=0.2019$ is reached, and integrating during the period of the simulation and dividing by the time lapse, an integrated efficiency of $\bar{\eta^{⁎}}=0.0944$ is reached. For a discretized in time system the integrated efficiency is calculated as:

(46) $$\bar{\eta^{⁎}}=\frac{1}{N_{steps}⁎\Delta t}\;\sum_{1}^{N_{steps}}\eta^{⁎}\cdot \Delta t$$

By analyzing Fig. 11, *c*) it can be seen that the mechanical efficiency takes negative values twice at every cycle. This is explained because when the device reaches one edge of the oscillation, it has zero speed and the rod is at its maximum deformation. And so, because of the elasticity of the rod, even with no force from the fluid, the VTW starts its displacement towards the other side. This means that the energy to move the device at the very end (or beginning) of the oscillation, comes from the elasticity of the rod.

The average output power, calculated with Eqs. (38) and (39), for a wind speed of U=9.1m/s is about 750 Watts. This seems a low power output when comparing with a small horizontal axes wind turbine, 4.3 meters of diameter ($14.5\;m^{2}$ swept area) available in the market Enair (2019) with a power output of 3kW at 9m/s of wind speed. Nevertheless, the analysis of the vorticity wind turbine in this study, is focused on representing the dynamics of the fluid and the elastic rod, where the modeling of the electrical generator is quite simple.

**Figure 12:**
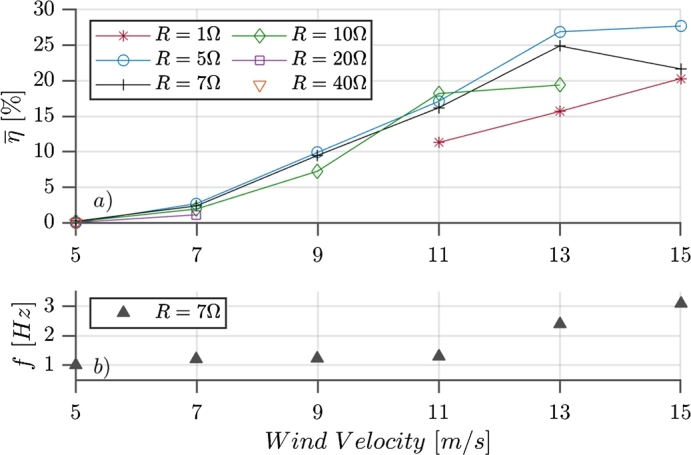
(a) Efficiency vs wind speed parametric in load resistance and (b) oscillation frequency vs wind speed using *R* = 7Ω.

The results shown so far, correspond to the same simulation, having a uniform flow velocity of $U_{o}=9.1\;m/s$, and two equal resistive loads of R=7Ω connected at each coil of the generator model. Several simulations were carried out varying alternatively the wind velocity and the resistive load. For each simulation the integrated efficiency was calculated and plotted in Fig. 12 (*a*) against the wind velocity. The different colors indicate different values of load resistance connected to the coils. Also, the frequency of the oscillation against the wind velocity is plotted in Fig. 12 (*b*) using 7Ω resistive loads.

The curves presented in Fig. 12 are generally continuous, displaying growing behavior. As the efficiency at low values of wind speed is almost zero, it improves the values for mid wind velocities (shown in the horizontal axis), and significantly higher values are reached for higher wind speeds. This is mainly for the structural damping of the rod. For low values of wind velocity, low forces are applied, and so the energy dissipated in the material is enough to keep the oscillations within a range of very small amplitude. When the lock-in effect is reached, the oscillations start to be wider, and thus higher values of efficiency are obtained.

By using different values of resistance the induced force can be regulated within certain limits (given by the other parameters of the generator), see Eq. (36). This means that the system damping, which includes the magnetic force, can be moderately modified by changing the resistance value. Furthermore, higher wind velocities produce higher forces acting on the mast, which result in higher transverse velocity that induce higher forces, opposite to the movement. All these phenomena, keeps the VWT oscillating within a reasonable amplitude around the vertical axis, and is why the lock-in effect can be maintained for higher values of wind speed.

Lower values of resistance produce larger magnetic forces, these are suitable for higher wind speeds. For instance, using R=1Ω (the red line in Fig. 12) gives an increasing efficiency for higher wind velocities, while the green, black and blue lines (R=10, 7 and 5 Ω respectively) starts to flatten in the same range. Simulations were performed until wind velocities of 15 m/s. For higher wind speeds, the forces, and therefore the displacement velocity of the device, start to be very high. Which requires a simulation domain with more control volumes and smaller time steps.

### 4.1 Convergence and sensitivity analysis

**Figure 13:**
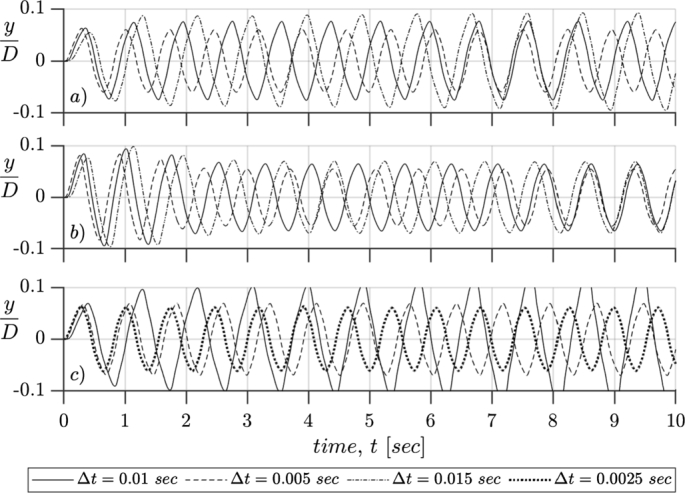
Sensitivity analysis for three mesh sizes (a) Δ*l* = 5 *cm*, (b) Δ*l* = 6.25 *cm* and (c) Δ*l* = 4 *cm*.

The results presented so far in this study were obtained with the Cartesian grid described in Fig. 1b, using cubic control volumes of size Δl=5 cm and a time step, Δt=0.01 seconds. However, several simulations were carried on to analyze the impact of varying the mesh size and the time step values. A summary of the mesh size convergence and time step sensitivity analysis is illustrated in Fig. 13. Panels *a*), *b*) and *c*) corresponds to cubic control volumes of 5, 6.5 and 4 cm respectively.

The mesh size and time step values were selected considering the amount of control volumes swept by the mast in a single time step. In this way, the amount of cells that the top of the mast sweeps in a single time step is limited between 0.25 and 1.8 at its maximum velocity.

In the upper panel of Fig. 13, plot *a*), the oscillation of the device is plotted against time, for three different simulations with the same domain (control volumes of Δl=5 *cm*) and varying the time step. There are slight differences in the amplitude and frequency of the motion. The smallest time step (Δt=0.005 *sec*) shows the smaller amplitude and higher frequency, while the higher amplitude and lower frequency is obtained with the largest time step (Δt=0.015 *sec*).

For the coarser mesh, panel *b*), the three time steps analyzed have almost negligible variations in the amplitude. However, for a 50% smaller time step (Δt=0.005 *sec*) the frequency of the oscillation is 7.5% higher, and for a 50% higher time step (Δt=0.015 *sec*) the frequency is 7.5% lower.

Finally, in the finest mesh, panel *c*), the simulation shows to be unstable for the same time step (Δt=0.01 *sec*), where the amplitude of the oscillations get larger in time. For this conditions the top of the mast sweeps more than 2 cells every time step, causing erratic and immeasurable forces over the mast that, when transmitted to the rod, induce physically unfeasible movement on it. This nonphysical behavior can be addressed by reducing the time step, simulations for Δt=0.005 and 0.0025 seconds present consistent behaviors.

### 4.2 Challenges

The results presented in the previous section describe a VWT adjusted to operate at a wind speed of $v_{wind}=9.1\;m/s$, seeking to maximize the output power, and thus, the efficiency of the device. However, very large deformations were found at very high wind speeds.

For very high wind speeds a more damped structure could be tested, as it would support larger forces. This can be achieved by changing the entire rod, for instance using a thicker one or changing the properties of the material (i.e. using one with higher structural damping), but this is not a functional option, as it would not be actually possible in a real physical setting to change the rod in order to accommodate the system for different wind speeds. A solution that would be both physically feasible and logistically sound would be to implement a variable damping system.

As the model of the generator implemented in these simulations can be interpreted as a damping force in the motion of the rod, by varying the parameters of the generator, the damping of the structure could be measurably modulated. The key is to find a set of parameters for the generator that can maintain the amplitude and velocity of the oscillations within a controlled range and at the same time maximize the output power. In this case, the resistive load connected to the circuit of the generator would be used to tune the damping, so as to analyze the behavior at different time steps. Equations (36) and (37) explain the inversely proportional relation between the magnetic force (damping) and the value of the load resistance. There is an available technology that could, after measuring the wind speed, automate the adjustment of the damping to optimize the power generation under each specific wind conditions.

Finally, although the displacement of the device is well captured for the transverse direction, it has to be taken into consideration that the study does not involve all the parameters of a real situation, as in this simulation the force in the along wind direction would not be transmitted to the rod, because the measurement of the displacement in this direction is not considered relevant for the study. Drag forces acting over the mast are quantitatively important, and they would produce larger displacements in the stream-wise direction, this effect has not been studied in this research. With respect to this, it is worth clarifying that the modeling of the generator that has been implemented is rather simple and performs well for linear displacements only. If the aim was to harvest energy from displacements in every direction, then another kind of power take off system should be considered, for instance a joystick-type generator that could generate with any angular displacement.

## 5 Conclusions and future work

The main objective of the present research was to analyze, through the use of mathematical models, the potential efficiency of a vorticity wind turbine. In the process of devising the tools to achieve that goal, a three-dimensional numerical method had to be developed for coupling the finite volume and discrete element methods in the analysis of flexible and elastic structures.

For the modeling of the VWT, bending moment transmission was incorporated to the already developed DEM applied to fishing nets in a previous work Sassi et al. (2017), whereas the elements of the fishnet are loose ropes, so there is no bending moment transmission in between the elements. Another important variation applied to the method was the approach used for integrating the equations of motion. In the case of the fishnet, a fourth order Runge-Kutta was implemented for solving the ordinary differential equations system, while for the VWT a centered finite difference, as the one proposed by Ivanov (2001), was used for the integration. It would be interesting, in order to improve the computational efficiency of the simulation, to incorporate a higher order method, such as the one used in Sassi et al. (2017), or even to consider the incorporation of an adaptive time step, a potential future development that was beyond the scope of this research.

The main findings of this work can be summarized in the following points:

• The three-dimensional coupled method for elastic and flexible structures seems to represent qualitatively well the dynamics of the VWT. The frequency of the oscillation (*f*) is practically the same to the vortex shedding frequency ($f_{vs}$), managing to capture the lock-in effect and the vortex shedding frequency is similar to the frequency calculated from the Strouhal number (*St*), tabulated for cylinders as a function of the Reynolds Number.
• The simulation for the implemented generator performs quantitatively well with values that are comparable in terms of peak and integrated efficiencies to the ones presented by Bernitsas et al., 2008, Bernitsas et al., 2009 in experimental physical tests for the VIVACE generator. Even taking into consideration that, at least in the simulation stage, the vorticity turbines are less efficient than current blade turbines, the practicality of the method in terms of cost and simplicity of the installation could prove it to be a convenient solution that complements rather well other conventional means.
• The conical shape of the mast was designed as a means to achieve a perfect synchronization of the vortex shedding all along the mast. Considering that the wind velocity increases with height in the atmospheric boundary layer, the diameter of the oscillating structure should also increase with height to favor the synchronization. The lock in effect was well captured in the simulations, and synchronization was achieved, along the height of the mast, for uniform flow.

In this research, only uniform flows have been tested, but it would be very enriching to simulate the efficiency of these devices under the dynamics of an atmospheric boundary layer. Also further research would allow determining the most efficient shape for the mast to optimize the synchronization of the vortex shedding along the height of the mast, and therefore maximize the output power.

It is worth mentioning the potential of the method to predict output power for this type of systems, because the ability to test different designs or types of applications would be easily implemented just by introducing the corresponding changes in the code. For instance, those incorporations could allow testing a more sophisticated power take-off system or even considering the adaptation of the device to produce energy underwater, harvesting the motion of waves and currents.

### Author contribution statement

Paolo Sassi: Conceived and designed the experiments; Performed the experiments; Analyzed and interpreted the data; Contributed reagents, materials, analysis tools or data; Wrote the paper.

Jorge Freiria: Analyzed and interpreted the data.

Mariana Mendina, Martin Draper, Gabriel Usera: Analyzed and interpreted the data; Contributed reagents, materials, analysis tools or data.

### Funding statement

This research did not receive any specific grant from funding agencies in the public, commercial, or not-for-profit sectors.

### Competing interest statement

The authors declare no conflict of interest.

### Additional information

Supplementary content related to this article has been published online at https://doi.org/10.1016/j.heliyon.2020.e05155.

## Supplementary material

HLY5155_from_source.pdf Three-dimensional view of the Simulation with the velocity field displayed in the x-direction over an horizontal plane at a height of 80% of the mast (animation). HLY5155_from_source.pdf

VWT_simulation.mp4 10 seconds of simulation of the vorticity wind turbine, with an uniform air flow of 9.1 m/s. Velocity Field in the x-direction at z=6.5 m., instantaneous output power of the generator and accumulated energy. VWT_simulation.mp4
