# Supplementary material for: Simulation of vorticity wind turbines
Source: Heliyon. 2020 Oct 9;6(10):e05155. doi: 10.1016/j.heliyon.2020.e05155 (PMC7557875; doi:10.1016/j.heliyon.2020.e05155)
Supplement: HLY5155_from_source.pdf — Three-dimensional view of the Simulation with the velocity field displayed in the x-direction over an horizontal plane at a height of 80% of the mast (animation). [file mmc1.pdf]

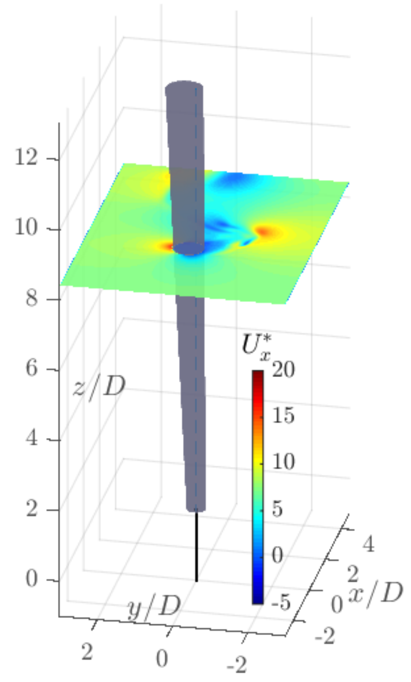

Figure 6: Three-dimensional view of the Simulation with the velocity field displayed in the x-direction over an horizontal plane at a height of 80% of the mast (animation).
